# Enhanced Protection against Ebola Virus Mediated by an Improved Adenovirus-Based Vaccine

**DOI:** 10.1371/journal.pone.0005308

**Published:** 2009-04-23

**Authors:** Jason S. Richardson, Michel K. Yao, Kaylie N. Tran, Maria A. Croyle, James E. Strong, Heinz Feldmann, Gary P. Kobinger

**Affiliations:** 1 Special Pathogens Program, National Microbiology Laboratory, Public Health Agency of Canada, Winnipeg, Manitoba, Canada; 2 Department of Microbiology, University of Manitoba, Winnipeg, Manitoba, Canada; 3 Department of Medical Microbiology, University of Manitoba, Winnipeg, Manitoba, Canada; 4 Department of Pediatrics and Child Health, University of Manitoba, Winnipeg, Manitoba, Canada; 5 Division of Pharmaceutics, College of Pharmacy, The University of Texas at Austin, Austin, Texas, United States of America; 6 Institute for Cellular and Molecular Biology, The University of Texas at Austin, Austin, Texas, United States of America; Institut Pasteur, France

## Abstract

**Background:**

The Ebola virus is transmitted by direct contact with bodily fluids of infected individuals, eliciting death rates as high as 90% among infected humans. Currently, replication defective adenovirus-based Ebola vaccine is being studied in a phase I clinical trial. Another Ebola vaccine, based on an attenuated vesicular stomatitis virus has shown efficacy in post-exposure treatment of nonhuman primates to Ebola infection. In this report, we modified the common recombinant adenovirus serotype 5-based Ebola vaccine expressing the wild-type ZEBOV glycoprotein sequence from a CMV promoter (Ad-CMVZGP). The immune response elicited by this improved expression cassette vector (Ad-CAGoptZGP) and its ability to afford protection against lethal ZEBOV challenge in mice was compared to the standard Ad-CMVZGP vector.

**Methodology/Principal Findings:**

Ad-CMVZGP was previously shown to protect mice, guinea pigs and nonhuman primates from an otherwise lethal challenge of *Zaire ebolavirus*. The antigenic expression cassette of this vector was improved through codon optimization, inclusion of a consensus Kozak sequence and reconfiguration of a CAG promoter (Ad-CAGoptZGP). Expression of GP from Ad-CAGoptZGP was substantially higher than from Ad-CMVZGP. Ad-CAGoptZGP significantly improved T and B cell responses at doses 10 to 100-fold lower than that needed with Ad-CMVZGP. Additionally, Ad-CAGoptZGP afforded full protections in mice against lethal challenge at a dose 100 times lower than the dose required for Ad-CMVZGP. Finally, Ad-CAGoptZGP induced full protection to mice when given 30 minutes post-challenge.

**Conclusions/Significance:**

We describe an improved adenovirus-based Ebola vaccine capable of affording post-exposure protection against lethal challenge in mice. The molecular modifications of the new improved vaccine also translated in the induction of significantly enhanced immune responses and complete protection at a dose 100 times lower than with the previous generation adenovirus-based Ebola vaccine. Understanding and improving the molecular components of adenovirus-based vaccines can produce potent, optimized product, useful for vaccination and post-exposure therapy.

## Introduction

Ebola virus (EBOV) is a member of the *Filoviridae* family causing a viral hemorrhagic fever with a lethality that can reach beyond 90% [1], [2]. While the precise mode of natural viral transmission to humans and nonhuman primates remains elusive, there are indications that bats may act as a primary source of infection [3]. EBOV causes hemorrhagic fever following virus entry in susceptible organisms where the virus appears to initially infect monocytes, macrophages, and dendritic cells leading to deregulated activation of innate immunity and a systemic inflammatory response syndrome. This results in massive destruction of critical organs, vascular damage and haemorrhage within 5–7 days post-exposure [4], [5]. Outbreaks of EBOV have primarily been localized to Central Africa with relatively low impact on human health worldwide, despite inflicting devastating consequences on affected communities. EBOV has however drawn increasing interest in the past years due to an increased number of natural human outbreaks and its potential use in biological warfare [6].

Human adenovirus serotype 5 (Ad) was initially developed as a delivery vehicle for therapeutic transgenes in a wide variety of animal models of genetic disease [7]. However, an inherent characteristic of recombinant Ad for gene therapy applications is the ability of the virus to elicit a strong immune response even in immunocompetent hosts, making Ad-based vectors attractive vaccine carriers [8]. Vaccination with Ad expressing the *Zaire ebolavirus* (ZEBOV) envelope glycoprotein (Ad-ZGP) has been shown to protect mice, guinea pigs and nonhuman primates from lethal ZEBOV challenges [9]–[11]. It is also currently being studied in a phase I clinical trial recently initiated in normal adults with the objective of evaluating safety and immune responses to the vaccine (http://clinicaltrials.gov/show/NCT00374309).

The use of attenuated vesicular stomatitis virus (VSV) vaccine platforms expressing the Ebola virus surface glycoprotein has also been useful for the treatment and protection of three animal models post-exposure [1], [12]. Administration of the VSV-based vaccine as late as 24 hours after lethal Ebola virus infection, resulted in 50% and 100% survival, in guinea pigs and mice respectively while 50% of rhesus macaques tested were protected when treated 20 to 30 minutes after exposure to Ebola [12]. While VSV appears to be one effective treatment strategy for Ebola infections, adenovirus-based vaccine may offer further useful characteristics such as the rapidity to produce large amounts of transgene which can promote a robust immune response soon after vaccination [1].

Viral regulatory elements such as the human cytomegalovirus immediate early gene (CMV) promoter induce high-level constitutive expression *in vitro* in a variety of mammalian cell lines and thus were largely used to generate many of the early gene transfer vectors [13]. Other technologies were developed in order to enhance gene expression notably for DNA vaccine platforms. In this context, codon optimization for translation in mammalian cells along with the hybrid CAG promoter which combines the human cytomegalovirus immediate early gene enhancer and a modified chicken beta-actin promoter were shown to improve protective immune responses after vaccination [14], [15]. Targeting of dendritic cells and high expression profiles are two major characteristics making recombinant adenovirus such a robust vaccine vector.

In this report, we modified the common recombinant adenovirus serotype 5-based Ebola vaccine expressing the wild-type ZEBOV glycoprotein sequence from a CMV promoter (Ad-CMVZGP) to enhance expression of the envelope antigen. The immune response elicited by this improved expression cassette vector (Ad-CAGoptZGP) and its ability to afford protection against lethal ZEBOV challenge in mice was compared on a dose-to-dose basis to that of the standard Ad-CMVZGP vector.

## Results

### Increased Ebola GP expression from an improved expression cassette adenovirus vector

**Figure 1:**
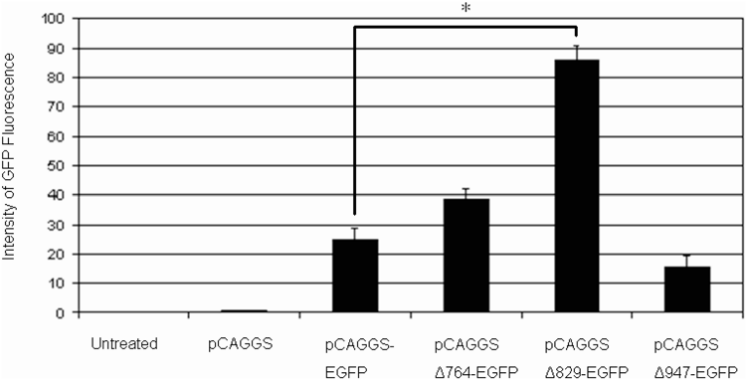
Expression intensity of EGFP reporter gene in transfected HEK 293T cells as determined by FACS. Portions of the 5′ untranslated region of pCAGGS downstream of the CAG promoter were systematically removed generating pCAGGSΔ764-EGFP, pCAGGSΔ829-EGFP and pCAGGSΔ947-EGFP. Assays were preformed in triplicate and repeated twice, the data shown is from one experiment. Error bars represent the standard deviation of the data. * p<0.001.

**Figure 2:**
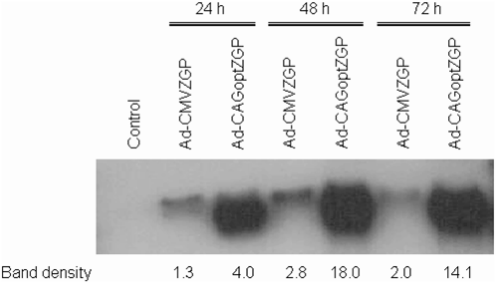
Western blot expression analysis of Ad-CMVZGP or Ad-CAGoptZGP. Proteins isolated from infected HEK 293 cells were separated by a 10% SDS PAGE then transferred to PVDF membrane. A mouse monoclonal anti-ZGP was used as the primary antibody and a goat anti-mouse horseradish peroxidase (HRP) conjugated antibody as the secondary antibody. 24, 48 and 72 hours indicate the time of total protein harvest post-infection. Band density corresponding to each lane is shown as determined by densitometry of the bands. The control represents untreated HEK 293 cells. An M.O.I. of 5 was used to infect the cells with either Ad-CMVZGP or Ad-CAGoptZGP for each time point. The preparation of Ad-CAGoptZGP or Ad-CMVZGP used had a non-infectious to infectious ratio of 73∶1or 19∶1 respectively.

The antigenic expression cassette of an E1/E3 deleted adenovirus serotype 5 vector was improved to provide enhanced expression of the Ebola GP. Improvements included codon optimization for translation in mammalian cells, inclusion of a consensus Kozak sequence and identification of a more efficient configuration of a CAG promoter. Portions of the 5′ untranslated region (UTR) of pCAGGS-MCS downstream of the CAG promoter were progressively truncated using endogenous restriction enzyme sites within the UTR sequence. The initial objective of systematically deleting portions of the 5′ UTR was to identify the minimal promoter region, capable of accommodating larger inserts for different applications. The CAGΔ829 deletion of the 5′ UTR resulted in a statistically significant substantial increase (p<0.001) in expression of an enhanced green fluorescent protein (EGFP) reporter gene compared to the full promoter containing EGFP (pCAGGS-EGFP) in transfected HEK 293T cells as determined by flow activated cell sorting (FACS) (Figure 1). The plasmid pCAGΔ829-EGFP repeatedly showed the highest intensity of EGFP and the regulatory element from the enhancer to the multiple cloning sites (MCS; named CAGα) was selected to drive the expression of the codon optimized Ebola GP in the adenovirus vector. The codon optimized ZGP was inserted downstream of the CAGα promoter in the adenovirus vector and Ebola GP expression was evaluated by western blot analysis following infection of HEK 293 cells at a multiplicity of infection (M.O.I.) of 5. An adenovirus encoding a CMV driven wild-type Ebola GP (Ad-CMVZGP) was used in parallel at the same M.O.I. for comparative analysis. Cell free extracts were recovered at 24, 48 and 72 hours post-infection and each separated by SDS-PAGE electrophoresis. Western blot analysis using a ZGP monoclonal antibody showed a single band corresponding to the predicted molecular weight of glycosylated ZGP at substantially higher levels in cell lysates infected with the Ad-CAGoptZGP than with the Ad-CMVZGP vector at each time point (Figure 2). Densitometry evaluation of the bands corresponding to ZGP revealed an average band density of 2.8 from the Ad-CMVZGP and of 18.0 from the Ad-CAGoptZGP vector at 48 hours post-infection. A marked increase of 3 to 7-fold was observed between 24 and 72 hours post-infection in samples infected with the Ad-CAGoptZGP vector when compared to Ad-CMVZGP. Similar ratios were observed from infected Vero E6 or MDCK cells with Ad-CAGoptZGP consistently expressing higher levels of ZGP than Ad-CMVZGP (data not shown).

### Enhanced T and B cell immune responses with low dose of Ad-CAGoptZGP

**Figure 3:**
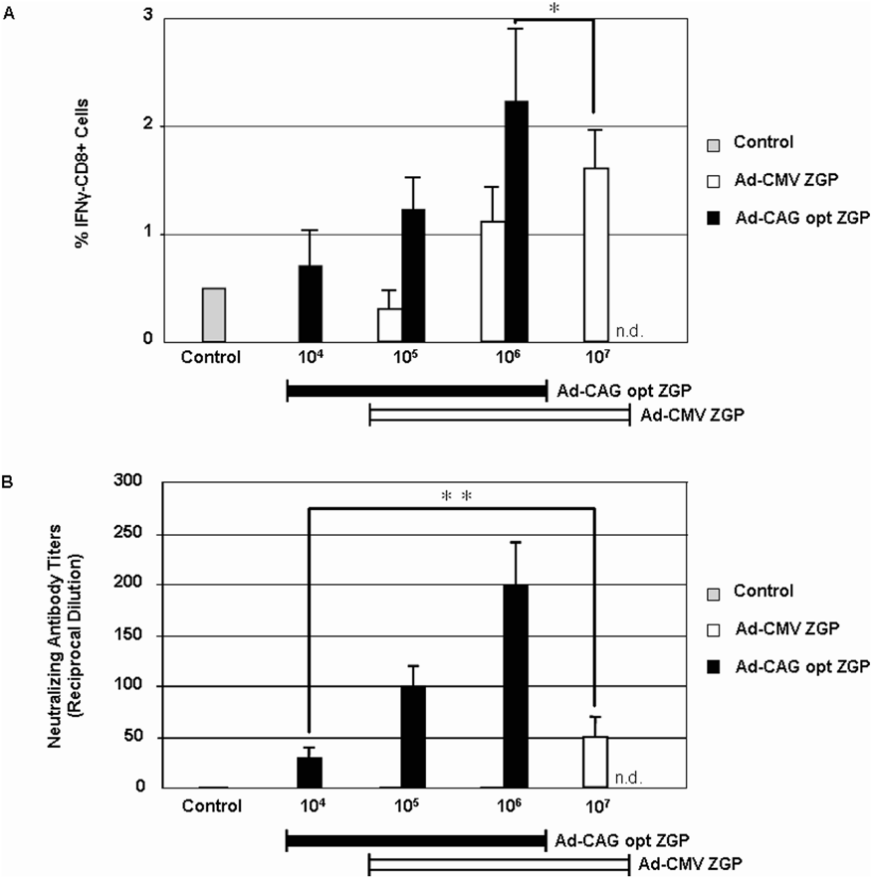
T and B cell responses following immunization. B10.BR mice were vaccinated I.M. with Ad-CMVZGP (1×10^5^, 1×10^6^ and 1×10^7^ IFU/mouse) or Ad-CAGoptZGP (1×10^4^, 1×10^5^ and 1×10^6^ IFU/mouse) and splenocytes were harvested 8 days later for A. IFN-γ CD8+ T cells frequency analysis or B. Neutralizing antibody (NAB) titers. Four to five mice were analyzed per group and the experiment was repeated twice. Levels of NAB to ZEBOV-EGFP were evaluated 25 days post-vaccination. Error bars represent the standard deviation of the data. n.d. refers to assays that were not done for Ad-CAGoptZGP at that IFU/mouse. * p = 0.0454; ** p = 0.1.

Stimulation of CD8+ T cells expressing IFN-γ was monitored following vaccination with Ad-CMVZGP or Ad-CAGoptZGP in mice. B10.BR mice were vaccinated I.M. with either Ad-CMVZGP at a concentration of 1×10^5^, 1×10^6^ or 1×10^7^ infectious forming units (IFU)/mouse or Ad-CAGoptZGP at a concentration of 1×10^4^, 1×10^5^ or 1×10^6^ IFU/mouse. The Ad-CAGoptZGP was administered over a lower spectrum of doses because of the higher expression profile of the ZGP antigen. The mice were sacrificed eight days post-immunization and the percentage of interferon-γ (IFN-γ)-CD8+ T cells was determined by FACS. Ad-CAGoptZGP at a dose of 1×10^5^ IFU/mouse elicited a frequency of 1.3±0.3% positive IFN-γ producing CD8+ T cells compared to 0.3±0.16% induced by Ad-CMVZGP at the same dose (Figure 3A). The frequency of positive IFN-γ producing CD8+ T cells increased to 2.2±0.7% for Ad-CAGoptZGP at 1×10^6^ IFU/mouse compared to 1.1±0.4% with the same dose of Ad-CMVZGP (Figure 3A). The frequency of IFN-γ+CD8 T cells with Ad-CMVZGP at 1×10^7^ IFU/mouse was at 1.6±0.4% in average (Figure 3A). Overall, the average frequency of positive IFN-γ producing CD8+ T cells was higher with 1×10^6^ IFU/mouse of Ad-CAGoptZGP than with 1×10^7^ IFU/mouse of Ad-CMVZGP although with a low statistical significance (p = 0.0454).

Neutralizing antibody was used to evaluate the B cell response in the serum of vaccinated animals. Sera from B10.BR mice were collected 25 days after immunization and assayed for the presence of neutralizing antibodies against ZEBOV expressing the enhanced green fluorescent protein reporter gene (ZEBOV-EGFP) [16]. Neutralizing antibodies were detected in sera obtained from Ad-CAGoptZGP immunized mice at all vaccine doses of 1×10^4^, 1×10^5^ and 1×10^6^ IFU/mouse with reciprocal dilutions ranging from 40±10 to 200±20 (Figure 3B). Mice immunized with Ad-CMVZGP had an average neutralizing antibody level of 50±20 reciprocal dilutions starting at the dose of 1×10^7^ IFU/mouse. Mice administered Ad-CAGoptZGP at 1×10^4^ IFU/mouse demonstrated neutralizing antibody levels similar to levels obtained from mice vaccinated with Ad-CMVZGP at 1×10^7^ IFU/mouse (p = 0.1).

### Immunization of mice with Ad-CAGoptZGP is protective at low doses against mouse-adapted ZEBOV

To determine the protective efficacy of Ad-CMVZGP versus Ad-CAGoptZGP vaccine vector, groups of 10 B10.BR mice were immunized with different doses of adenovirus vector and challenged 28 days later with a lethal dose (LD_50_ = 1000) of mouse-adapted ZEBOV [17]. All control mice vaccinated with phosphate buffered saline (PBS) died by day 9 post-challenge whereas all mice vaccinated with doses of 1×10^4^, 1×10^5^ and 1×10^6^ IFU/mouse of Ad-CAGoptZGP were fully protected with no weight loss or other clinical signs of disease (Table 1). Protection was complete in mice vaccinated with Ad-CMVZGP at 1×10^6^ and 1×10^7^ IFU/mouse but was only partially protective at 1×10^5^ IFU/mouse (Table 1). Mice vaccinated with the lower dose of 1×10^5^ IFU/mouse had 60% mortality and up to 17% weight loss in surviving animals (Table 1).

**Table 1:** Survival and weight loss of mice challenged with ZEBOV 28 days post-vaccination.

Vaccine	Concentration (IFU/Mouse)	Survival (Percentage)	Weight Loss (Percentage)
Ad-CMVZGP	1×10^7^	100	0
Ad-CMVZGP	1×10^6^	100	0
Ad-CMVZGP	1×10^5^	40	17
Ad-CAGoptZGP	1×10^6^	100	0
Ad-CAGoptZGP	1×10^5^	100	0
Ad-CAGoptZGP	1×10^4^	100	0
Control	na^1^	0	>25

Eight to nine B10.BR mice were used in each group.

1 not applicable.

### Infected mice survive a lethal challenge with mouse-adapted ZEBOV when vaccinated soon after exposure

**Figure 4:**
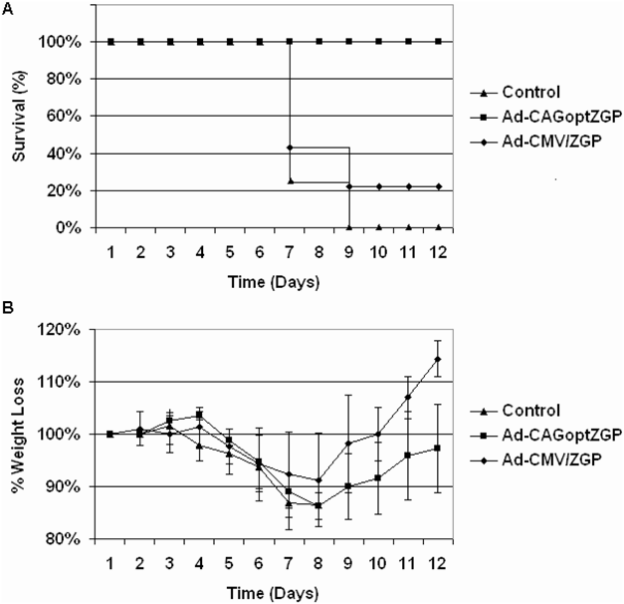
Protective efficacy following intramuscular (I.M.) immunization 30 minutes post-challenge. Groups of nine to eleven B10.BR mice were challenged with 1000×LD_50_ of mouse-adapted ZEBOV and vaccinated I.M. 30 minutes later with a single dose of 5×10^7^ IFU of Ad-CAGoptZGP or Ad-CMVZGP per mouse. Preparations of Ad-CAGoptZGP or Ad-CMVZGP had non-infectious to infectious ratio of 73∶1 or 19∶1 respectively. Error bars represent the standard deviation of the data. Data represent A. percent survival and B. percent body weight loss over time. Control mice were PBS-treated 30 minutes after challenge. This experiment was repeated once with B10.BR mice and once with BALB/c mice and generated similar results.

The substantial improvement in protection and enhanced immune responses observed with low dose of the Ad-CAGoptZGP vaccine raised the question whether this vaccine vector could afford post-exposure protection against ZEBOV in mice. To answer this question, B10.BR mice were challenged with 1000×LD_50_ of mouse-adapted ZEBOV and vaccinated I.M. 30 minutes later with either Ad-CAGoptZGP or Ad-CMVZGP at a concentration of 5×10^7^ IFU/mouse. The challenge was uniformly lethal in PBS control mice and 22% of mice treated with Ad-CMVZGP survived although weight loss was observed (Figure 4A and B). In contrast, 100% of the mice administered Ad-CAGoptZGP survived the challenge however weight loss of up to 14% was also observed (Figure 4A and B).

**Figure 5:**
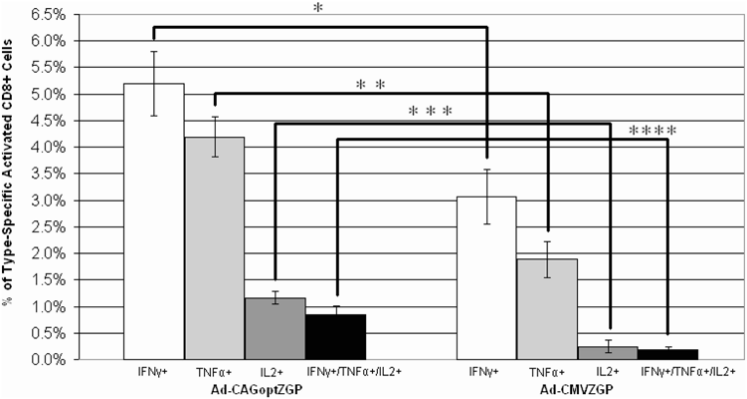
T cell frequency analysis at day 6 post-immunization. Groups of 4 B10.BR mice were vaccinated I.M. with 1×10^8^ IFU/mouse of Ad-CMVZGP or Ad-CAGoptZGP and splenocytes were harvested 6 days later, re-stimulated with the peptide TELRTFSI, and production of IFN-γ, TNF-α, and Il-2 from CD8+ T cells was monitored by FACS. Error bars represent the standard deviation of the data. The experiment was repeated once and showed similar results. * p<0.001; ** p<0.001; *** p<0.01; **** p<0.01.

One of the possible mechanisms contributing to post-exposure protection is the induction of an immune response reaching protective levels before Ebola virus can kill the host. Immune responses were monitored at day 6 after vaccination with each vector to assess the levels of activated T cells and neutralizing antibody detectable just before mice are expected to succumb to a mouse-adapted Ebola challenge of 1000×LD_50_ (between 7 to 10 days post-challenge). Stimulation of CD8+ T cells expressing IFN-γ, TNF-α and/or IL-2 following Ad-CMVZGP or Ad-CAGoptZGP was evaluated after I.M. vaccination with 1×10^8^ IFU/mouse. Immunized mice were sacrificed 6 days post-immunization and the percentage of IFN-γ, TNF-α and/or IL-2 CD8+ T cells were determined by FACS. Ad-CAGoptZGP vaccinated mice had a frequency of 5.2±0.604%, 4.2±0.379% or 1.2±0.121% positive IFN-γ, TNF-α or IL-2 producing CD8+ T cells respectively. In comparison, Ad-CMVZGP had a frequency of 3±0.521%, 1.9±0.340% or 0.25±0.118% positive IFN-γ, TNF-α or IL-2 producing CD8+ T cells respectively (Figure 5). Triple positive CD8+ T cells expressing IFN-γ, TNF-α and IL-2 were detected in 0.85±0.154% of the total from Ad-CAGoptZGP vaccinated mice compared to 0.2±0.055% in total CD8+ T cells isolated from Ad-CMVZGP immunized mice (Figure 5). The frequencies of IFN-γ, TNF-α or IL-2 producing CD8+ T cells individually or combined (triple positive) were increased with statistical significance from mice immunized with Ad-CAGoptZGP compared to Ad-CMVZGP (p<0.001, p<0.001, p<0.01, p<0.01 respectively). Neutralizing antibody assay performed on B10.BR mice sera collected 6 days after immunization revealed levels of 16+/−9 reciprocal dilution following vaccination with Ad-CAGoptZGP whereas no neutralizing antibodies were detected in sera of Ad-CMVZGP immunized mice (data not shown).

## Discussion

Vaccine vectors derived from adenoviruses have been shown to be efficacious in a number of animal species showing susceptibility to different infectious agents [18], [19]. However, acute toxicity has also been reported with the administration of high doses of this virus [20]. The present study systematically analyzed the effect of increasing antigenic expression from an adenovirus-based vaccine on immune responses and protective efficacy in mice against mouse-adapted ZEBOV. Technologies mainly evaluated and adopted by the DNA vaccine community including gene optimization was used to develop the described vector for use against ZEBOV. The improved Ad-CAGoptZGP vaccine vector was evaluated side-by-side with the commonly used CMV driven wild-type Ebola GP-encoding adenovirus which has shown protective efficacy in nonhuman primates against ZEBOV [21].

The human cytomegalovirus immediate early gene (CMV) promoter induces high-level constitutive expression of encoded genes *in vitro* and *in vivo* in a variety of cell types [13], [22], [23] and therefore has been incorporated into many recombinant adenovirus-based vaccines. The present study indicates that an optimized antigenic expression cassette including the modified CAG promoter and codon optimization can substantially improve immune responses and protection generated by an adenovirus-based vaccine. The optimized Ad-CAGoptZGP vaccine strengthened immune responses at a doses 10 to 100-fold lower than those commonly used for the Ad-CMV driven Ebola GP vaccine. The optimized vaccine also fully protected mice against a lethal challenge with mouse-adapted ZEBOV at a dose 100 times lower than the minimal dose required to achieve full protection with the first generation adenovirus vaccine. Understanding the relative contribution of each molecular determinant (i.e. promoter, Kozak sequence, codon optimization etc.) individually may help design future optimized adenovirus-based as well as other sub-unit vaccines. These experiments are currently underway.

Overall, the substantial improvement in vaccine efficacy can be useful for many applications. The same protective efficacy can be achieved with lower doses of adenovirus vector allowing for a more extended use of each preparation and importantly minimizing toxicity from the vaccine vector. Alternatively, protection can be achieved with doses equivalent or even lower than for example the lowest dose of 1×10^9^ virus particles currently evaluated in clinical trials [http://www.clinicaltrials.gov/ct/show/NCT00374309?order=1].

Despite the fact that weight loss was noted, complete survival of mice infected with mouse-adapted ZEBOV was observed following administration of a relatively low dose (5×10^7^ IFU/mouse) of the improved vaccine 30 minutes after challenge, supporting the concept of post-exposure induced protection. Although, the precise mechanism remains to be fully elucidated, a higher number of IFN-γ, TNF-α and IL-2 positive CD8 T cells were detected from splenocytes of Ad-CAGoptZGP immunized mice 6 days post-vaccination when compared to Ad-CMVZGP immunized mice. Levels of NAB were also detected at day 6, but only from mice vaccinated with the improved Ad-CAGoptZGP vector. This suggests that in addition to the T cell response, B cells may also play a role in increasing survivability in mice. However, antibodies alone may not be sufficient as a small percentage of mice vaccinated with Ad-CMVZGP survived despite the lack of detectable NAB. These observations would be in agreement with the concept that the improved adenovirus vaccine could stimulate a more rapid immune response which reaches protective levels before ZEBOV could cause irreversible damage to the host. Although similar post-exposure protection against different filoviruses has been reported using the VSV vaccine platform [12], [24] extending this important characteristic of post-exposure efficacy from a replication competent filovirus vaccine to a replication defective vaccine vector such as those currently being evaluated in several clinical trials and newer optimized versions such as these should be valuable. Overall, these results support further evaluation of the improved Ad-CAGoptZGP vaccine in other animal models of ZEBOV infection such as guinea pigs and nonhuman primates.

## Materials and Methods

### Construction of the optimized expression cassette

A minimal CAG (chicken-β-actin promoter and cytomegalovirus enhancer) sequence was identified from plasmid pCAGGS-MCS following the sequential deletion of the 5′ non-translated region in pCAGGS-EGFP expressing the enhanced green fluorescent protein (EGFP). Three truncated versions of pCAGGS-EGFP were generated using the restriction enzymes *Psp*OMI/*Xba*I (Δ764 base pairs), *Eco*47III/*Xba*I (Δ829 base pairs) or *Eco*47111*/Acc*65I (Δ947 base pairs) that cut within the UTR sequence. The digested DNA was filled in with Klenow fragments (NEB Inc., Ipswich, MA) then ligated with T4 DNA ligase generating pCAGGSΔ764-EGFP, pCAGGSΔ829-EGFP and pCAGGSΔ947-EGFP or pCAGβ, pCAGα, and pCAGγ, respectively. Transfection of wild-type pCAGGS-EGFP, pCAGα, pCAGβ or pCAGγ into human embryonic kidney cell line (HEK 293T) was performed with the lipid-based Effectene transfection system according to the manufacturer recommendations (Qiagen, Mississauga, Ontario).

### Gene synthesis of human codon-optimized Ebola GP

The ZEBOV GP sequence (Genbank/NCBI; Mayinga strain 76 accession number AF086833 protein accession number Q05320) used contains an additional adenosine in the editing site for the production of full length GP which is subject to post-translational modifications (cleavage, glycosylation; N-linked and O-linked carbohydrates) [25]. The GP sequence was codon-optimized for translation in mammalian cells generating optZGP for increased antigenic expression from the adenovirus-based vaccine. Based on NCBI human codon usage databases, the wild-type ZEBOV GP sequence uses 36% of codons most frequently found in mammalian cells. OptZGP was further codon optimized to 70% by choosing the most abundant and/or second most abundant codons. The optZGP was synthesized from 40-mer oligonucleotides and then inserted into the pCAGα expression vector. Briefly, 121 oligonucleotide primers (Operon Biotechnologies, Inc., Huntsville, Alabama) each 40 nucleotides in length with an overlap of 20 nucleotides were generated to encode the full length codon-optimized optZGP open reading frame (ORF). The oligonucleotides were then combined in a three cycle PCR protocol to produce a complete double stranded optZGP using a method previously described [26]. A consensus Kozak sequence was also introduced into the forward primer of the optZGP to facilitate optZGP expression. The final amplification of the codon-optimized optZGP was accomplished by PCR using iProof High Fidelity Polymerase (BioRad) and the construct was confirmed by sequencing. The full length opt ZGP was then cloned downstream of pCAGα.

### Construction and production of adenoviral vectors

The molecular clone of E1/E3-deleted adenovirus vector expressing the optimized pCAGα-optZGP expression cassette was inserted into pShuttle using the restriction enzymes *Spe*I and *Nhe*I and then cloned directly into the E1 region of human adenovirus serotype 5 (BD Adeno-X expression system I, BD Biosciences, Palo Alto, CA). The CMV driven wild-type sequence of ZGP in the same adenovirus vector was used for comparison. The authenticity of each vector was confirmed by sequencing and the recombinant virus was rescued by transfecting the linearized DNA into HEK 293 cells maintained in Dulbecco's modified Eagle's medium (DMEM) supplemented with 1% penicillin, 1% streptomycin, 1% L-glutamate, 1% sodium pyruvate, and 10% fetal bovine serum. Large-scale infections (5×10^8^ cells) were initiated from positive transfectants and purified by cesium chloride as previously described [10]. Genome structures of vectors were analyzed by restriction digestions of viral DNA and compared with those of the molecular clones as previously described [10]. Particle number and infectivity of vectors were determined by standard optical density and immunodetection of the hexon protein, respectively, following infection of HEK 293 cells with limiting dilutions of each vector preparation according to the recommendations by the manufacturer (Adeno-X rapid titer kit, Clontech, Mountain View, CA). Several Ad-CAGoptZGP and Ad-CMVZGP preparations were generated and quantified for both infectious particle and total particle number. Preparations with a ratio below 1∶200 infectious to total particle were used in this study.

### Expression of Ebola ZGP and optimized ZGP

HEK 293 cells were cultured in 6-well plates to approximately 80% confluence. The cells were infected with recombinant adenoviral vectors Ad-CMVZGP or Ad-CAGoptZGP at a M.O.I. of 5. The M.O.I. used for Ad-CMVZGP or Ad-CAGoptZGP on HEK 293 cells was based total infectious particles per ml as determined by immunocytochemistry against the hexon protein (Adeno-X rapid titration kit, Clonetech, Mountain View, CA). Preparations of Ad-CAGoptZGP or Ad-CMVZGP used for expression analysis had a non-infectious to infectious ratio of 73∶1 or 19∶1 respectively. The analysis was repeated with a preparation of Ad-CAGoptZGP with non-infectious to infectious ratio of 90∶1 or Ad-CMVZGP with ratio of 128∶1 (not shown). At 24 hours, 48 hours, and 72 hours after infection cell supernatants were removed from the tissue culture well and 50 µL of 2× radioimmunoprecipitation (RIPA) buffer (10 mL Triton X-100; 10 g lauryl sulfate; 5 mL 10% SDS; 30 mL 5 M NaCl; 20 mL 1 M Tris pH 7.7; 20 mL 0.5 M EDTA pH 8.0) was added per 1 cm^2^ surface area of tissue culture well. Cell lysates were then collected, normalized by total protein content and analyzed under reducing conditions with 5× SDS-gel loading buffer (50 mM Tris-HCl pH 6.8; 100 mM dithiothreitol; 2% (w/v) electrophoresis grade SDS; 0.1% bromophenol blue; 10% (v/v) glycerol on a 10% SDS-PAGE. Following electrophoresis, proteins were transferred by electroblotting to a PVDF membrane (Bio-Rad) and revealed with a mouse immune serum to Ebola ZGP at a 1∶1500 dilution as the primary antibody followed by a horseradish peroxidase-conjugated goat anti-mouse secondary antibody diluted 1∶7500. Immunodetection (Amersham ECL system, Piscataway, NJ) was performed according to the manufacturer. To determine band density proteins were transferred by electroblotting to Immobilon PVDF membrane (Millipore). The membrane was blocked overnight in 100% Sea-Block (Fisher) and then washed with PBS-Tween (0.01%). A mouse anti Ebola-GP monoclonal antibody was used as the primary antibody with a 1∶1500 dilution and incubated for one hour. Antibody against β-actin was used as a control at a dilution of 1∶7500. The membrane was then washed with PBS-Tween (0.01%) followed by the addition of Cy5 secondary antibody (Cedarlane) at a dilution of 1∶100 for one hour. Scanning was accomplished using the Typhoon 9410 and quantification using ImageQuant TL v2002.1.

### Animal models, vaccination and challenge

B10.BR mice ((MHC H-2^K^), The Jackson Laboratory, ME), a strain in which a dominant CD8 epitope for ZGP has been identified [27], were used to evaluate protection as well as B and T cell immune responses against ZGP. The T cell response was analyzed by evaluating the frequency of CD8+ T cells positive for IFN-γ production or cell division upon peptide stimulation by FACS as previously described [10]. Groups of 8 to 11 B10.BR mice were immunized with adenovirus vectors at a dose ranging from 1×10^4^ to 1×10^7^ IFU per animal 28 days before challenge. Mice were also vaccinated with Ad-CAGoptZGP at 5×10^7^ IFU/mouse 30 minutes post-challenge. All protective and post-exposure vaccines used in this study were derived from one of two preparations of either Ad-CAGoptZGP or Ad-CMVZGP. Preparations of Ad-CAGoptZGP had non-infectious to infectious ratio of 73∶1 and 90∶1. Ad-CMVZGP preparations had ratio of 19∶1 and 128∶1. Vaccination was performed intramuscularly (I.M.) with 50 µl of recombinant adenoviral vector diluted in PBS in each posterior hind limb. Mice were challenged by intraperitoneal injection with 1000×LD_50_ in 200 µl of mouse-adapted ZEBOV [17]. After challenge, the animals were weighed every day for 12 to 16 days and monitored for clinical signs using an approved scoring sheet. All procedures and the scoring sheet were approved by the Institutional Animal Care Committee at the National Microbiology Laboratory (NML) of the Public Health Agency of Canada (PHAC) according to the guidelines of the Canadian Council on Animal Care. All infectious work was performed in the ‘Biosafety Level 4’ (BSL4) facility at NML, PHAC.

### Neutralization assay

Sera collected from immunized mice were inactivated at 56°C for 45 minutes and serial dilutions of each sample (1∶10, 1∶20, 1∶40, etc, in 50 µl of DMEM) was mixed with equal volume of ZEBOV expressing the EGFP reporter gene (ZEBOV-EGFP) [12] (100 transducing units/well, according to EGFP expression) and incubated at 37°C for 90 minutes. The mixture was then transferred onto subconfluent VeroE6 cells in 96-well flat-bottomed plates and incubated for 5–10 minutes at room temperature. Control wells were infected with equal amounts of ZEBOV-EGFP without addition of serum or with non-immune serum. 100 µl of DMEM supplemented with 20% FBS was then added to each well and plates were incubated at 37°C in 5% CO_2_ for 48 hours. Cells were subsequently fixed with 10% buffered formalin for 24 hours and examined under a fluorescent microscope. The total number of EGFP positive cells were counted in each well and sample dilutions which showed >50% reduction in the number of green cells compared to controls scored positive for neutralizing antibody. All infectious *in vitro* work was performed in the BSL4 laboratory of NML, PHAC.

### Frequency of IFN-γ, TNF-α and IL-2 positive CD8+ cells

Splenocytes or mononuclear cells were isolated by grinding spleen tissues in L-15 medium and purifying cells through filtration followed by 3 washes with PBS and resuspension in RPMI 1640. Isolated splenocytes were pooled from 4 B10.BR mice per experimental group 6 and 8 days post-immunization and added to microwells along with the TELRTFSI [28] peptide which carries the ZGP immunodominant MHC class I epitope for mice of the H-2^k^ haplotype (B10.BR). Control cells were incubated either without peptide or with the nonspecific stimulator, SEB (200 ng/ml). For the evaluation of IFN-γ, TNF-α or IL-2 positive CD8+ T cells, splenocytes (1×10^6^/sample) were cultured for 5 hours at 37°C in 96-well round bottom microtiter plate wells in DMEM supplemented with 10% FBS, 10^−6^ M 2-ME and GolgiStop (BD PharMingen, San Diego, CA) at 1 µl/ml as described previously [10]. Briefly, stimulated cells were stained with a FITC-anti mouse CD8a (BD PharMingen) at 1∶300 dilution followed by a PE-anti mouse IFN-γ antibody (BD PharMingen) diluted 1∶150 or PECY7-anti mouse TNF-α antibody (BD PharMingen) diluted at 1∶100 or APC-anti mouse IL-2 antibody (BD PharMingen) diluted at 1∶100. Stained cells were run through a LSRII flow cytometer, acquiring at least 300,000 events per sample. Final data analyses were performed using the software Flowjo (Ashland, OR). A response was considered positive when the frequency from stimulated samples was over twice that of non-stimulated cells or those stimulated with unrelated peptides. Samples for the ELISPOT assay and the evaluation of IFN-γ positive CD8+ T cells were tested in duplicate and each experiment repeated twice.

### Statistical analysis

Data were analyzed for statistical difference by performing one-way analysis of variance (ANOVA, Tukey-Kramer multiple comparisons test) or repeated measure ANOVA when appropriate. The differences in the mean or raw values among treatment groups were considered significant when p<0.05.
